# Retrospective study of the effect of disease progression on patient reported outcomes in HER-2 negative metastatic breast cancer patients

**DOI:** 10.1186/1477-7525-9-46

**Published:** 2011-06-20

**Authors:** Mark S Walker, Murad Hasan, Yeun Mi Yim, Elaine Yu, Edward J Stepanski, Lee S Schwartzberg

**Affiliations:** 1 ACORN Research, LLC, 6555 Quince, Suite 400, Memphis, TN 38119, USA; 2 Genentech, 1 DNA Way, Mailstop 66, South San Francisco, CA 94080, USA; 3 West Clinic, 100 N Humphreys Blvd., Memphis, TN 38120, USA

## Abstract

**Background:**

This retrospective study evaluated the impact of disease progression and of specific sites of metastasis on patient reported outcomes (PROs) that assess symptom burden and health related quality of life (HRQoL) in women with metastatic breast cancer (mBC).

**Methods:**

HER-2 negative mBC patients (n = 102) were enrolled from 7 U.S. community oncology practices. Demographic, disease and treatment characteristics were abstracted from electronic medical records and linked to archived Patient Care Monitor (PCM) assessments. The PCM is a self-report measure of symptom burden and HRQoL administered as part of routine care in participating practices. Linear mixed models were used to examine change in PCM scores over time.

**Results:**

Mean age was 57 years, with 72% of patients Caucasian, and 25% African American. Median time from mBC diagnosis to first disease progression was 8.8 months. Metastasis to bone (60%), lung (28%) and liver (26%) predominated at initial metastatic diagnosis. Results showed that PCM items assessing fatigue, physical pain and trouble sleeping were sensitive to either general effects of disease progression or to effects associated with specific sites of metastasis. Progression of disease was also associated with modest but significant worsening of General Physical Symptoms, Treatment Side Effects, Acute Distress and Impaired Performance index scores. In addition, there were marked detrimental effects of liver metastasis on Treatment Side Effects, and of brain metastasis on Acute Distress.

**Conclusions:**

Disease progression has a detrimental impact on cancer-related symptoms. Delaying disease progression may have a positive impact on patients' HRQoL.

## Introduction

Breast cancer is the most common cancer among women in the United States (excluding skin cancer) and the second leading cause of cancer death in women (second to lung cancer)[1]. Although disease incidence in women decreased between 1999 and 2005, the incidence of advanced disease has remained stable[2].

Few women (6%) diagnosed with breast cancer are initially diagnosed with distant metastases. Of those who are, 27.1% are alive after 5 years,[3] a rate considerably lower than the 5-year survival rate for women diagnosed with regional (84%) or localized (98%) disease[4]. In addition to those with advanced disease at diagnosis, approximately 30% of patients diagnosed with earlier stage disease will eventually develop metastases[5]. Given the high incidence of breast cancer, these data suggest that a substantial and growing number of women diagnosed with advanced disease will experience an extended period of survival.

Treatment of metastatic breast cancer (mBC) may be associated with physical symptoms and emotional distress that adversely impact patients' physical functioning, psychological well-being, and social support systems--all dimensions of quality of life. Studies have also suggested that health related quality of life (HRQoL) is positively associated with subsequent survival duration,[6] making it an important consideration in treatment decisions. While the relationship between HRQoL and survival is not well understood, other research has shown that patients provided with accurate, relevant information regarding potential treatment side effects, symptom burden and HRQoL during treatment showed decreased emotional distress and anxiety, and an enhanced ability for self care, [7,8] clear benefits of understanding the impact of treatment.

Although there is a significant body of literature evaluating HRQoL in women with breast cancer,[9,10] much of this work has been focused on survivorship,[11-13] women treated in adjuvant settings,[14-16] or in women with recurrent disease[17-19]. HRQoL research with mBC patients has occurred primarily in the clinical trial setting,[20-22] has included multiple cancer types,[23] and has focused on characterizing HRQoL at either a single point in time or at a couple of fixed time points[23,24]. Although these studies may examine HRQoL within different lines of therapy,[21] they generally do not assess the impact of disease progression itself, and are constrained in their ability to characterize the trajectory of HRQoL over time.

The primary goal of this research was to evaluate the impact on HRQoL of disease progression in general, and of specific sites of metastasis, in a HER-2 negative mBC population. An additional goal was to identify those symptoms most often reported as severe at baseline in this population. We focused on HER-2 negative patients because they comprise the bulk of women with breast cancer and because we believe that the HRQoL trajectory may be different in women with HER-2 positive disease. We hypothesized that disease progression would be associated with a decrease in composite indicators of HRQoL and worsening of symptom burden related to fatigue, pain, and other high frequency symptoms. We also hypothesized that metastasis to different organ systems would differentially affect self reported patient reported outcomes (PROs).

## Methods

### Patients and Setting

This was a retrospective chart review and database analysis conducted at seven geographically distributed community oncology practices in the United States. Patients were eligible if they were female, at least 18 years of age, had a confirmed diagnosis of stage IV breast cancer, were HER-2 negative, had experienced at least one disease progression after diagnosis with mBC per physician note, had at least 180 days of follow up post progression, and had completed at least one Patient Care Monitor (PCM) assessment, described further below, in the 60 days both prior and subsequent to the first disease progression.

### Procedures

Potentially eligible patients were identified by community oncology practices affiliated with ACORN Research, and medical charts reviewed to determine final study eligibility. Completed case report forms were submitted via dedicated facsimile to the ACORN analysis center and entered into a secure database. Institutional review board approval was obtained from IntegReview in Austin TX.

### Study Measures

The primary endpoints for this study were indices of symptom burden and HRQoL as collected by the PCM. PCM, version 2.0, is an 86-item self-report measure that asks patients to rate the severity of symptoms on an 11 point (0 to 10) Likert-type scale, where higher scores reflect more severe symptoms. Patients are instructed to rate items to describe "how bad the symptom has been for you during the past week, including today." The items are generally a single word or short phrase, such as: "Fatigue, tiredness or weakness," or "Rash" for physical symptoms; "I am sad or depressed," for psychological symptoms; and "I am generally able to function normally" for Physical functioning.

The PCM is administered via touch screen tablet PC as a routine part of care at participating community oncology practices, and takes 10 - 12 minutes to complete after the first administration. The PCM produces standardized scores (T scores) for six screening scales: General Physical Symptoms, Treatment Side Effects, Despair and Depression, Acute Distress, Impaired Ambulation, and Impaired Performance. The PCM has been shown to be valid for assessing HRQoL in cancer patients and has been used in a number of studies[25-29]. Demographic, disease, and treatment variables were also collected, and their impact on PCM items and index scores was assessed. These variables are listed in Tables 1 and 2 and described in the statistical analysis section.

**Table 1:** Demographic and Treatment Characteristics (N = 102)

Variable	N (%)
*Age (Mean, SD, Median)*	57.0 (13.5) 57
*Ethnicity*	
Caucasian	73 (71.6%)
African American	26 (25.5%)
Other	2 (2.0%)
Unknown	1 (1.0%)
*BMI (Mean, SD, Median)*	29.0 (6.5) 28.0
*Had Prior Breast Cancer Surgery*	89 (87.3%)
*Had Prior Radiation Therapy*	73 (71.6%)
*Had Prior Neoadjuvant Therapy*	16 (15.7%)
*Had Prior Adjuvant Therapy*	63 (61.8%)
*First Line Therapy*	
None Reported	4 (3.9%)
Hormonal Therapy Only	30 (29.4%)
Taxane Based Therapy	43 (42.0%)
Non-Taxane Based Therapy	25 (25.0%)
Had Toxicity Related Discontinuation	7 (6.9%)
*Received Second Line Therapy*	92 (90.1%)
Had Toxicity Related Discontinuation	6 (6.5%)

**Table 2:** Disease Characteristics (N = 102)

Variable	N (%)	
*Stage at Diagnosis*		
Stage I	12 (11.8%)	
Stage II	33 ( 32.4%)	
Stage III	20 ( 19.6%)	
Stage IV	26 (25.5%)	
Unknown	11 (10.8%)	
*ER Positive*	76 (74.5%)	
*PR Positive*	55 (53.9%)	
*ECOG PS*		
< 2	34 (33.3%)	
= 2	6 ( 5.9%)	
> 2	3 (3.0%)	
Unknown	59 ( 57.8%)	
*# Months from initial dx to metastasis (Mean, SD)*	37.9 (42.3)	
*# Months from metastasis to first progression (Mean, SD)^a^*	13.9 (14.8)	
*Had second disease progression*	78 (76.5%)	
*# Months from 1st to 2nd progression (Mean, SD)*	6.6 (5.4)	
*Deceased Per Medical Record*	45 (44.1%)	
*Metastatic Sites*	*At initial Metastatic Diagnosis*	*At First progression*
Bone	61 (59.8%)	67 (65.7%)
Brain	0 (0.0%)	2 (2.0%)
Liver	27 (26.5%)	34 (33.3%)
Lung	29 (28.4%)	36 (35.3%)
Peritoneum	6 (5.9%)	6 (5.9%)
Other	35 (34.3%)	44 (43.1%)
*Comorbidities Present (N,%)*		
Hypertension	49 (48%)	
CHF	2 (2.0%)	
History of MI or Stroke	4 (3.9%)	
Other CVD	26 (25.5%)	
Diabetes	18 (17.6%)	

*^a^*Median time from metastases to disease progression = 8.8 months

### Statistical Methods

Descriptive statistics were generated for all study variables. Linear mixed models were employed to examine change in PCM index scores over time, controlling for individual, disease and treatment characteristics. Methods followed those described by Littell et al. and Cnaan et al.[30,31] Interval since diagnosis (Interval) was modeled as a random effect, using restricted maximum likelihood estimation. The covariance matrix of random effects was specified as unstructured in each model.

Each model examined whether PCM scores were collected before or after disease progression (Progression), and also examined the proximity of PCM survey date to the date of the first disease progression (Proximity). Proximity was modeled as logarithmic, except as noted, to reduce its redundancy with Interval. Because they were fundamental to the questions under study, each model included Interval and Progression irrespective of statistical significance. Analyses also examined interactions of Progression with Proximity and Interval.

The models examined several other variables, and retained these if significant or nearly so. These include the effect of being on vs. off chemotherapy at the time of a PCM assessment, and having vs. not having metastatic disease at each of several key sites at the time of a PCM assessment (bone, brain, liver, lung, and peritoneum). Each model also examined several patient-level variables: 1) age, 2) race, 3) body mass index (BMI), 4) stage of disease at initial diagnosis, 5) baseline ECOG performance status (PS; 0 - 1 or missing vs. 2 - 4). Models also examined first-line treatment regimen, coded in three groups as taxane-based chemotherapy vs. non-taxane-based chemotherapy vs. hormone therapy only or no treatment. First line treatment regimen was included only if significant. As a result, the effect of regimen is reported for some models but not others. Finally, we also examined whether first-line treatment regimen contained bevacizumab, and included this variable where significant.

Results are summarized in Table 3 for all PCM endpoints, and presented graphically for two PCM index scores. Note that although disease progression occurred after different intervals for different patients (a factor modeled within each analysis), it is shown at the median interval of about 9 months in the figures. In addition to the reporting of statistical significance for effects within each model, effects were also described relative to the minimal important difference (MID) of the effects[32,33]--the smallest change in HRQoL that is important to the patient. This value is 0.5 to 1 point for individual PCM items, and 1.5 to 3 points for PCM index scores[34].

**Table 3:** Summary of Mixed Model Results for PCM Items and PCM Index Scores

	Mixed Model Results			
	
Endpoint	Change over time since Diagnosis (Interval)	Change at Disease Progression (Progression)	Change with Proximity to Progression (Proximity)	Main or Interaction Effects involving Regimen Group*^a^*
**PCM Items**				

Fatigue	Improving (p = .02)	Worsened (p = .004)	No effect	Progression: H = NT > T (p = .052)^b^
Pain	No effect	Worsened (p = .051)	No effect	No effect
Trouble Sleeping	No effect	No effect	No effect	Main effect: H = NT < T (p = .001)
**PCM Index Scores**				

General Physical Symptoms	Improving (p < .001)	Worsened (p < .001)	Tended to worsen leading into progression, with less improvement leading out (p = .088)	No effect
Treatment Side Effects	Improving (p = .013)	Worsened (p < .001)	No main effect	Proximity: H = NT < T (p = .005) [Taxane had worse scores distal from progression]
Acute Distress	Improving (p = .002); improving faster after progression (p = .022)	Worsened (p = .002)	No effect	No effect
Despair and Depression	No effect	No effect	No effect	No effect
Impaired Ambulation	No effect	No effect	No effect	No effect
Impaired Performance	Improving (p = .003)	Worsened (p = .003)	No effect	Main effect: H < NT (p = .005) > T (p = .004)

*^a^*H = Hormone therapy or no therapy, NT = Non-taxane based regimen, T = Taxane based regimen. Main effect = main effect of Regimen group; Progression = interaction of regimen group with Change at Progression; Proximity = interaction of regimen group with Change with Proximity to Progression. ^b^P values are for specific contrasts involving regimen groups, with Taxane and Hormone only groups tested against the Non-taxane, which was the reference condition. P values for overall regimen effects are reported in the text where significant.

Analyses were conducted with SPSS Version 15.0. All statistical tests were interpreted at alpha = .05, two tailed, and no adjustment was made for multiple comparisons.

## Results

### Sample Development

**Figure 1:**
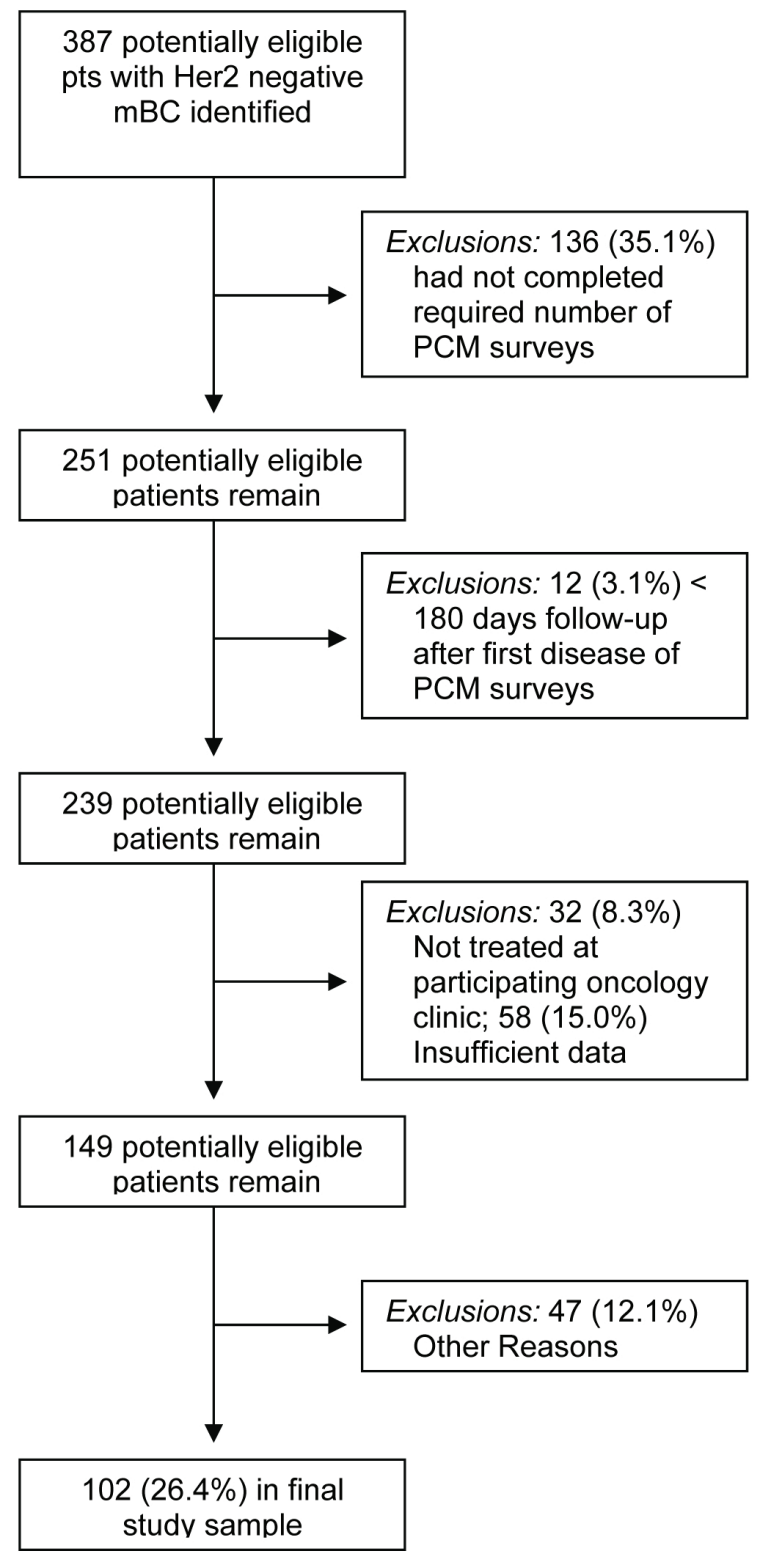
**Flow Diagram of Study Sample Development**.

A total of 387 potentially eligible women with stage IV, HER-2 negative disease and at least one disease progression post mBC diagnosis were identified. Of these, 136 (35.1%) were ineligible due to insufficient PCM data, primarily because the PCM was not uniformly administered at all clinics. Another 15% of patients were excluded because of insufficient medical chart data, 8.3% because they received follow-up care at a non-participating clinic, 3.1% because of inadequate follow-up (i.e., < 180 days) after disease progression and 12.1% for other reasons. The remaining 102 patients were deemed study eligible, and represent the final study sample. Figure 1 depicts sample development graphically.

### Demographic, Disease and Treatment Characteristics

The sample was largely Caucasian (71.6%) or African American (25.5%) with a mean age of 57.0 (13.5) years. All subjects were women with stage IV HER-2 negative breast cancer. The median time from metastasis to first disease progression was 8.8 months (range: 1.1 to 74.9 months). Additional information regarding demographic, disease, and treatment characteristics is reported in Tables 1 and 2.

### Patient Care Monitor Assessment

There were 1698 valid PCM assessments available from 101 patients in the study (mean = 16.1 assessments per patient, SD = 14.0). Data from one patient was excluded due to incompleteness. Of the 1698 PCM surveys, 46.4% were completed after the first disease progression, and 81.8% were completed while patients were on chemotherapy.

#### Baseline Symptom Severity

Baseline symptom severity was of interest as an indicator of the symptom burden with which patients begin chemotherapy, and of the specific symptoms that tend to be a problem. Baseline was defined as the interval between metastatic diagnosis and start of chemotherapy for this assessment. Fifty-four baseline PCM observations were available from 39 patients. Item ratings were dichotomized for assessment of baseline symptom severity, with ratings of 7 or higher indicating severe symptoms. Of particular interest for this study were those PCM items that contribute to the General Physical Symptoms and Treatment Side Effects index scores, and that were severe at baseline in 10% or more of cases. Items from these indices, and the frequency with which they were rated as severe at baseline, included fatigue (15.0%), physical pain (17.5%), and trouble sleeping (31.3%).

#### Linear Mixed Models Analysis of PCM Items

Linear mixed models analysis of the fatigue, tiredness and weakness PCM item showed a small but significant effect of Interval (p = .02), with scores decreasing (improving) by about 1/3 point per year. Though significant, this effect falls below the MID (≥ 0.5 points) over the course of one year. ECOG PS 2 - 4 was associated with scores 1.7 points higher (worse) than PS 0 - 1 (p = .035). There was also a significant interaction of first-line treatment regimen with Progression, in which patients in the taxane group showed almost no increase in fatigue scores at progression (about 0.1 points), whereas other patients saw scores increase 0.7 - 0.8 points at progression.

Linear mixed models analysis of the PCM pain item showed that pain ratings were lower (better) by 0.6 points during chemotherapy (p < .001). Pain scores remained flat over time, but increased about 0.3 points at progression, an effect that approached statistical significance (p = .051) but that was not clinically relevant. The presence of bone metastasis was a significant predictor (p = .008), with scores about 1 point higher with bone metastasis present. This effect was superimposed on the more modest effect of progression itself, and may underestimate the combined effect of disease progression which involves spread of disease to the bone.

PCM data for the trouble sleeping item showed no effects of Interval or Progression. Results did show a significant effect of race (p = .045), in which Caucasian patients reported sleep scores 1.7 points lower (better) than minority patients. There was also an effect of lung metastasis (p = .025), with scores about 1.5 points higher (worse) when lung metastasis was present. In addition, there was a main effect of first-line treatment regimen (p = .004), in which patients on taxane regimens had scores on trouble sleeping that were more than 3 points higher (worse) than those on non-taxane regimens. Mixed model results for Fatigue, Pain, and Trouble Sleeping are summarized in Table 3.

#### General Physical Symptoms

**Figure 2:**
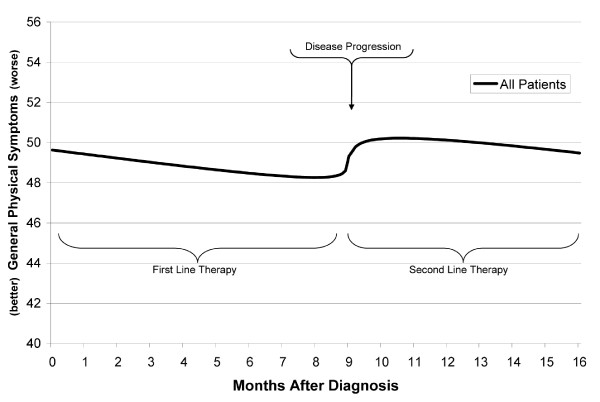
**Linear Mixed Model of General Physical Symptom Index Scores**.

Linear mixed model analysis of the General Physical Symptoms index score showed a significant effect of Interval (p < .001), with scores improving by 1 point every 4 months. As noted, the MID for PCM index scores is between 1.5 and 3 points. This indicates that the improvement seen over 6 months is a clinically relevant effect. Scores worsened at Progression by 2.4 points (p < .001), but there was also a near significant interaction of Progression with Proximity (p = .088). As shown in Figure 2, the nature of the interaction was that scores increased by about 2 points at progression, and resumed gradual improvement from the elevated level. ECOG PS 2 - 4 (vs. PS 0-1 or missing) was associated with an increase of 7.4 points in symptom severity (p = .016; not shown). Being on a bevacizumab containing regimen was also significant (p = .025), and was associated with a 5.9 point decrease in symptom severity (not shown).

#### Treatment Side Effects Symptoms

Analysis of the Treatment Side Effects index scores showed a significant effect of Interval (p = .013), with index scores decreasing (improving) about 1.5 points over a 9 month period. There was also a significant effect of Progression (p < .001), with scores increasing 2.3 points at progression. Patients with ECOG PS 2 - 4 had scores nearly 7 points higher (worse) than patients with PS 0-1 or missing (p = .002). Scores were nearly 2 points higher on chemotherapy vs. off (p < .001). The scores of patients who had brain or liver metastasis at the time of the survey were 5.4 and 2.7 points higher, respectively, than scores of patients without the specific metastasis (p = .02 and p = .001, respectively). Although the effect of brain metastasis is large, it should be noted that only 1 - 2% of PCM surveys were collected from patients with brain disease, compared with 27% from patients with liver metastasis.

**Figure 3:**
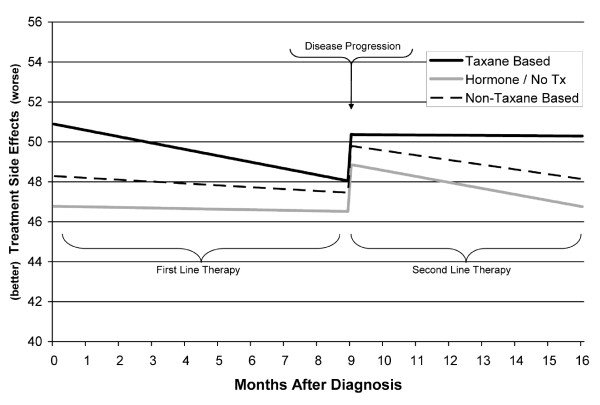
**Linear Mixed Model of Treatment Side Effects Index Scores**.

Results also showed a significant interaction between treatment regimen and Proximity (p < .001; with Proximity modeled as linear). The interaction, shown in Figure 3, shows scores for the taxane group were higher early in first line treatment than for the other regimen groups, and were stable rather than improving after progression.

#### Psychological Symptom Measures

Results for analysis of Acute Distress showed a significant effect of Interval (p = .002), with scores improving by about 1 point every 4 months. The main effect of Progression was significant, and reflected a 2.3 point increase in scores at disease progression. Interval interacted with Progression (p = .022), in that the rate of improvement in scores increased very slightly after disease progression. The effect of chemotherapy was also significant (p = .042), with scores just over 1 point lower (better) when patients were on vs. off chemotherapy. There was also a significant effect of brain metastasis (p = .033), with scores about 8 points higher when brain metastasis was present.

Linear mixed model analysis of Despair and Depression index scores showed almost no effects. Scores were stable over time, and increased less than 1 point at disease progression--both nonsignificant effects. ECOG PS, however, was significant (p = .021), with ECOG PS 2 - 4 associated with an increase of 5.8 points in symptom severity.

#### Physical Functioning Measures

Linear mixed models analysis of Impaired Ambulation index scores showed no significant effects of either Interval or Progression. However, there was a significant effect of ECOG PS (p = .001), with ECOG PS 2 - 4 associated with an increase of 9.6 points in symptom severity compared with ECOG PS 0-1 or missing. There was also a counterintuitive effect of lung metastasis (p = .008), with scores 3.3 points lower (better) when lung metastasis was present. The explanation for this effect is unknown.

Analysis of Impaired Performance showed a significant effect of Interval (p = .003), with scores improving by about 1 point every 4 months. The effect of Progression was also significant (p = .003), with a 1.7 point increase in scores at progression. After progression, scores resumed the pattern of gradual improvement. There was also a significant effect of treatment regimen (p = .006), with patients on non-taxane based therapies scoring nearly 7 points higher (worse) than patients on other therapies. ECOG PS was again significant (p = .001), with ECOG PS 2 - 4 associated with scores that were 12.1 points higher (worse) than patients with ECOG 0-1 or missing. Mixed model results for all of the PCM index scores are summarized in Table 3.

## Discussion

The results of this retrospective research suggest that disease progression in patients with HER-2 negative mBC is associated with a modest but significant worsening of General Physical Symptoms, Treatment Side Effects, Acute Distress and Impaired Performance scores. The effects were not significant for Despair and Depression or for Impaired Ambulation. It is plausible that controlling for sites of metastasis attenuated the estimated effect of disease progression for outcomes in which a specific site of metastasis (e.g. liver, brain) was significant. Although the effects of disease progression appear less pronounced than corresponding effects observed in previous study of breast cancer patients in the adjuvant breast cancer treatment setting,[28] most were statistically significant and reflect clinically relevant symptoms or adverse effects.

By following patients with mBC through two lines of therapy, this study provides an understanding of the trajectory of HRQoL over time, and quantifies the impact of disease progression across different HRQoL domains. Since the current study examined HRQoL with repeated assessments collected as a routine part of clinical care in a real-world, community oncology setting, findings may be more generalizable to the larger population than a single or limited domain studies conducted as part of a clinical trial.

We hypothesized that there would be deleterious effects of disease progression evident on both PCM index scores and individual PCM symptoms. Such effects were observed across many, though not all, of the PCM indices and items examined in this study, and are generally consistent with previous research[23].

The effects of disease progression that were statistically significant also appeared clinically relevant (i.e., ≥ MID), though smaller than the effects of disease recurrence observed among adjuvant cancer patients,[28] and smaller than some other effects examined in this study (particularly performance status). This attenuated effect should not be surprising, in that progression in the metastatic setting may not represent the same change of status as progression in the adjuvant setting, in which a patient may have previously thought herself cured.

In addition to general effects of disease progression, there were significant deleterious effects of specific sites of metastatic disease on the Treatment Side Effects and the Acute Distress index scores, and on the trouble sleeping PCM item. The mechanism by which specific sites of metastasis affect the outcomes is not clear, but likely varies across site of metastasis and HRQoL domain.

Although not a primary focus of the study, we did examine the effect of first line treatment regimen, particularly taxane vs. not-taxane based treatments. Taxane treatment is associated with significant toxicities, including peripheral neuropathy, and joint or muscle pain[35]. Interference with other activities, including increased sleep problems such as reported in a recent Canadian study,[36] and as reported in this study, may reflect secondary effects of these toxicities. As shown in Figure 3, patients on taxane therapies in the first line appear to report somewhat elevated treatment side effects. The overall effect of treatment regimen was nonsignificant, but the taxane group did appear to have more severe symptoms early in the first line treatment period. This pattern would appear to reflect adaptation to treatment side effects over the course of first line therapy. In contrast to this, patients on non-taxane chemotherapy had significantly worse Impaired Performance index scores. Heightened symptoms for the non-taxane group were not observed for other PCM endpoints, and may reflect selection of patients with existing performance impairment into non-taxane treatment regimens.

Findings from this study may be useful to clinicians in several ways. First, results of this study affirm that disease progression in mBC patients has broad impacts, affecting patients across multiple domains of HRQoL. Beyond this, however, it is important to note that the magnitude of the effects of progression tended to be modest. Although the overall clinical picture for mBC patients may be very serious, many of these patients appear to have adapted to their medical situation, and to be relatively nonreactive to adverse clinical events. This was especially evident in the psychological functioning domains, in which patients experienced distress from which they rebounded after progression, and in which the progression event appeared to not even register in Despair and Depression index scores. Clinicians should also note that patients with poor PS had poorer HRQoL. Although this is not surprising, it should be noted that the effect of ECOG PS represents the most consistent and pronounced effect on PROs observed in this study, an effect generally several times that of disease progression. This information may be useful to clinicians as they weigh the tradeoffs between efficacy, toxicity, patient status, and quality of life in their choices of treatment of mBC.

There were several limitations to this study. First, although data were collected from a number of geographically distributed oncology practices, the study used a convenience sample that may differ in unknown ways from the underlying population. Second, the study was retrospective, and therefore limited by the availability of existing data in the assessment of HRQoL. Third, although the number of assessments available for analysis was relatively large, the number of patients from whom these were drawn was more modest. Finally, we examined patients with metastatic breast cancer treated in community practice settings. Results may therefore not generalize to patients with other diseases, with disease at other stages, and to patients treated in other settings.

## Conclusions

Despite these limitations, the clear conclusion of this study is that progressive disease in HER-2 negative women with metastatic breast cancer is associated with clinically relevant worsening of symptoms across multiple domains, and that symptom severity may partly depend on the organ systems affected by metastatic disease. Although this study was not interventional, these findings suggest that delaying disease progression may have a beneficial effect on the health related quality of life of patients with metastatic breast cancer.

## Competing interests

The study reported in this paper was funded by Genentech, Inc. Portions of this research were presented at the 2009 meeting of the San Antonio Breast Cancer Symposium. YY and EY are employed by Genentech, and receive stock or stock options from Roche. They declare that they have no other competing interests. The other authors declare that they have no competing interests.

## Authors' contributions

MW participated in the design of the study, carried out the statistical analysis, participated in the interpretation of findings, and contributed to manuscript development. MH coordinated all data collection and participated in the interpretation of study findings. YY and EY participated in the design of the study, the interpretation of findings, and contributed to manuscript development. ES and LS participated in the design of the study and the interpretation of findings. All authors read and approved the final manuscript.
